# Prospective of ^68^Ga Radionuclide Contribution to the Development of Imaging Agents for Infection and Inflammation

**DOI:** 10.1155/2018/9713691

**Published:** 2018-01-04

**Authors:** Irina Velikyan

**Affiliations:** ^1^Section of Nuclear Medicine and PET, Department of Surgical Sciences, Uppsala University, Uppsala, Sweden; ^2^Preclinical PET Platform, Department of Medicinal Chemistry, Uppsala University, Uppsala, Sweden; ^3^PET Centre, Centre for Medical Imaging, Uppsala University Hospital, 75185 Uppsala, Sweden

## Abstract

During the last decade, the utilization of ^68^Ga for the development of imaging agents has increased considerably with the leading position in the oncology. The imaging of infection and inflammation is lagging despite strong unmet medical needs. This review presents the potential routes for the development of ^68^Ga-based agents for the imaging and quantification of infection and inflammation in various diseases and connection of the diagnosis to the treatment for the individualized patient management.

## 1. Introduction

The blossom of ^68^Ga utilization is reflected in the continuous rapid growth of the number of basic and clinical research publications as well as clinical trials and clinical practice [1–3]. The potential scope of ^68^Ga-based imaging agents is rather extensive including ligands specifically targeting receptors, enzymes, and antigens; hapten and effector molecules involved in pretargeted imaging; small molecules with biological function to monitor glycolysis, hypoxia, cell proliferation, and angiogenesis; nontargeting particles of various sizes for imaging of ventilation and perfusion [4]. The leading clinical application area is oncology with targeted imaging of somatostatin receptors (SSTR), prostate specific membrane antigen (PSMA), integrin receptors, glucagon-like peptide 1 receptors (GLP1R), gastrin-releasing peptide receptors (GRPR), human epidermal growth factor receptor family (HER2), and pretargeted imaging of carcinoembryonic antigen (CEA) [1, 3, 5].

The scope of ^68^Ga-based imaging agents for inflammation and infection is rather limited despite disease diversity and magnitude, and strong unmet medical need [1, 4, 6]. However, the research and development of ^68^Ga-based tracers for the diagnosis and discrimination of inflammation and infection accelerated during last five years [7–19]. Such ^68^Ga-based tracers with specific action could also considerably contribute to drug development. Unfortunately, the failure rate of new therapeutic drugs, in general, is rather high and it is a costly process. PET offers advantages such as possibility of quantifying the target occupancy by the drug very early in the development in vivo in humans due to the microdosing concept thus facilitating stratification of candidate therapeutic drugs.

This review presents the status of the ^68^Ga-based imaging agents for inflammation and infection and discusses the potential routes for the development of the agents and their connection to the treatment for the individualized patient management.

## 2. Infection and Inflammation

Infection is caused by the invasion of such pathogens as bacteria, virus, fungi, parasite, or prion. It is a significant cause of morbidity and mortality globally, especially in children causing more death than any other disease. Tuberculosis, malaria, and AIDS stand for about 50% of all lethal cases claiming 5 million lives and causing 300 million illnesses each year. Bacterial infection, for example, tuberculosis and multidrug resistant bacteria, presents diagnostic and therapeutic challenges [20, 21]. Inflammation is immune response to microbial invasion or an injury and can either be related to the pathogens or be sterile. It can be classified as acute or chronic, and the latter has been investigated as the major cause of inflammatory autoimmune, cardiovascular, neurological, and cancerous diseases.

In order to control infectious diseases and provide efficient treatment, early diagnosis as well as discrimination between bacterial and sterile inflammation is crucial. The disease specificity of the diagnostic tools is a desirable characteristic. Currently available diagnostic means present some disadvantages. Clinical laboratory tests such as white blood cell (WBC) counts and C-reactive protein (CRP) cannot unambiguously distinguish between bacterial and viral infection and may result in unnecessary treatment with antibiotics [22]. Radiological imaging techniques such as magnetic resonance imaging (MRI), X-ray, computed tomography (CT), and ultrasound are morphological and rely on the anatomical changes that occur at later stage of the disease. Moreover, these methods are not specific to neither inflammation nor infection type. Detection of viral infection is even more challenging since it does not produce anatomic changes as bacterial infection does even when the viral infection is severe.

In contrast to morphological imaging techniques, functional methods such as gamma scintigraphy (Single Photon Emission Computed Tomography (SPECT) and planar gamma imaging) and Positron Emission Tomography (PET) provide fast, whole-body, and noninvasive real time evaluation of physiology and pathology on molecular level early in disease processes before noticeable changes in anatomical structure occur. The whole-body examination might be of great importance especially in cases of occult infection [23]. The respective examinations can be repeated in order to monitor the treatment outcome resulting in personalized medicine approach [24–27]. The advantages of PET over SPECT are intrinsic to the technology and are presented with higher examination throughput, considerably higher sensitivity, possibility of detection, and quantification of tracer picomolar amounts as well as tracer uptake kinetics recording and dynamic image reconstruction [28]. In recent years, the stand-alone PET scanners have been substituted with hybrid PET-CT scanners that offer both high sensitivity of functional PET and temporal/spatial resolution of morphological CT in one examination. The hybrid PET-MRI scanners have also entered market providing advantages of MRI over CT in higher soft tissue contrast and absence of radiation dose to the patient. PET has demonstrated efficiency and profitability in individualized patient diagnostics especially in oncology, and its impact on patient management has been recognized by Medicare and Medicaid Services [29].

### 2.1. Common Clinical Imaging Agents for Inflammation and Infection

There are a few radiopharmaceuticals used in clinical routine, and they are nonspecific in their action: ^67^Ga-Citrate, ^99m^Tc/^111^In-white blood cells (WBC), and [^18^F]-fluorodeoxyglucose ([^18^F]FDG) [30]. They target components of inflammatory response to injury and infection and accumulate in the lesions as a result of an increased blood flow and enhanced vascular permeability. ^67^Ga-Citrate presumably transfers ^67^Ga to transferrin and lactoferrin that accumulate at the inflammation site on the cells such as leukocytes and B-lymphocytes expressing respective receptors [31]. Moreover, ^67^Ga can be accumulated in the macrophages, bacteria, and fungi via siderophores. Radiolabelled WBCs accumulate in the sites of leukocyte infiltration and do not discriminate infective from sterile inflammation [32]. [^18^F]FDG accumulates in leukocytes, macrophages, monocytes, lymphocytes, and giant cells due to upregulation of glucose transporters [33].

^67^Ga-Citrate has been in clinical use for imaging of infection and inflammation for over 40 years. It is applicable, for example, for the diagnosis of lung infections, acute/chronic osteomyelitis, tuberculosis, sarcoidosis, and retroperitoneal fibrosis [34]. However, the specificity of the agent is suboptimal with accumulation in malignancies and bone remodeling sites as well as bowel excretion pathway. Moreover, radiation doses to the healthy organs and tissues are unfavorable and the examination requires several visits to the hospital with an interval of 1–3 days between radiopharmaceutical administration and examination.

Radiolabelled autologous WBCs have been used for a wide range of infections such as peripheral osteomyelitis, postoperative infection, joint prosthesis infection, diabetic foot infection, cardiovascular infection, fever of unknown origin (FUO), opportunistic infection, central nervous system infection, musculoskeletal infection, and inflammatory bowel disease for over three decades. Various labelling techniques using ^111^In-oxine, ^99m^Tc-sulfur colloids, and ^99m^Tc-exametazime (HMPAO) have been developed; however the radiopharmaceutical preparation procedure is complicated and potentially hazardous for both personnel and patient [21, 30]. Moreover, the examination process is very demanding on the patient [35].

Most nuclear medicine applications worldwide (90%) stand for diagnostics with leading position for ^99m^Tc-based radiopharmaceuticals, especially in cardiology [36]. The most essential contribution to the improvement of the patient management in oncology has been presented by [^18^F]-fluorodeoxyglucose ([^18^F]FDG)/PET-CT reflecting the elevation of glucose transporter expression in tumour cells, and providing nearly universal application in the evaluation of various fast growing cancer types. [^18^F]FDG/PET-CT stands for over 90% of all PET-CT examinations [37, 38]. [^18^F]FDG/PET is an established diagnostic means also in infection and inflammation, and the major indications for it are FUO, sarcoidosis, peripheral bone osteomyelitis, suspected spinal infection, metastatic infection, bacteremia, and vasculitis [33]. However, demand for the imaging agents towards disease specific targets in cancer and inflammation/infection is growing [39, 40] since [^18^F]FDG fails to detect slowly growing tumours and to discriminate malignancy from sterile inflammation, infection, wound healing, tuberculosis, sarcoidosis, and reactive lymph nodes [41, 42]. Another disadvantage is high accumulation of [^18^F]FDG in healthy organs such as brain and gut resulting in suboptimal image contrast and consequently potential risk for lesion detection failure.

### 2.2. Unmet Medical Need

Noninvasive and specific diagnosis of many inflammatory diseases such as sarcoidosis, osteomyelitis, inflammatory bowel disease, and rheumatoid arthritis as well as early and accurate diagnosis of deep-seated infectious diseases such as septic arthritis, abscesses, endocarditis, and infections of prosthetics and implants would benefit patients [20]. Introduction of specific imaging agents disclosing cellular mechanisms of various diseases on molecular level would allow improvement in patient management and treatment outcome. There is a strong need for specific imaging agents not only for the accurate and quantitative diagnosis but also for the prognosis, treatment selection, planning, and adjustment as well as response monitoring as, for example, requirement for a certain antibiotic and treatment duration. Moreover, the imaging could guide surgical procedures and monitor implants of medical devices or transplanted organs [43]. Such imaging guided treatment would decrease the cost, side effects, and overtreatment avoiding immune suppression effects in inflammation and possibly reducing the problem of antimicrobial resistance by the termination of an accomplished successful treatment as early as possible. There are potential challenges in targeting both components of inflammatory response and microbes specifically: discrimination between infectious and sterile inflammation; discrimination between acute and chronic inflammation; discrimination between various infectious microorganisms; discrimination between pathogenic bacteria and microbiota; targeting specific types of bacteria; difficulty of accessing bacteria aggregated in a biofilm; and quantification of reproducing bacteria.

Health care requires further improvement of efficiency, safety, and quality of treatment with patient personalized approach that would allow early diagnosis which is a crucial factor in the reduction of mortality and patient management cost [81]. The concept of individualized patient management on molecular level with regard to both diagnostics and therapy is based on discoveries and success in genomics, proteomics, and biotechnology. Those achievements also accelerate the development of various imaging agents, and the application of molecular imaging diagnostic techniques is expanding very fast globally contributing considerably to the realization of personalized medicine.

## 3. Advantages of ^68^Ga: Nuclide Properties and Chemistry

Such radionuclides as ^11^C, ^18^F, ^64^Cu, ^68^Ga, ^89^Zr, ^99m^Tc, ^111^In, and ^124^I are used in various radiopharmaceuticals for diagnostic imaging with PET and SPECT (Table 1). With regard to PET, ^18^F stands for 41%, ^11^C stands for 31%, and ^64^Cu, ^68^Ga, ^89^Zr, and ^124^I stand for 28% of the radiopharmaceuticals [82]. With regard to SPECT, ^99m^Tc and ^111^In stand, respectively, for 42% and 29% of the radiopharmaceuticals. As mentioned above in the field of inflammation and infection gamma emitting ^67^Ga, ^99m^Tc, ^111^In, and positron-emitting ^18^F are commonly in use. The choice of a radionuclide depends on various aspects of production and application: availability, production mode, and cost of the radionuclide; nuclear characteristics and decay mode of the radionuclide; labelling chemistry pathways and duration; radiation dose to subjects; relevance of the physical half-life of the radionuclide to the pharmacokinetic time frame of the imaging agent. Within the group of gamma emitters used for SPECT, the production via generator system is an advantage that contributes to the leading position of ^99m^Tc due to ready accessibility and lower cost. Moreover, the single and lower gamma energy of ^99m^Tc results in higher image resolution as compared to ^67^Ga and ^111^In and shorter half-life of ^99m^Tc reduces radiation dose to the patient (Table 2).

The advantages of PET such as higher spatial resolution, sensitivity, and accurate signal quantification are crucial, especially in the case of small size lesions. Furthermore, dynamic scanning allows modeling and investigation of the mechanism of the interaction between the imaging agent and target. Even though ^68^Ga has a relatively high positron energy, the resolution of the images is comparable to that of ^18^F, since it is the scanner detector resolution (4–6 mm) which is the limiting factor [4, 83, 84]. The 68-min half-life of ^68^Ga is not compatible with ligands of slow pharmacokinetics, for example, antibodies. Thus other positron emitters such as ^124^I, ^89^Zr, and ^64^Cu with longer half-lives allowing 2–4 days required for the clearance of the agent for the blood circulation and washout for the nontarget tissue are more relevant. The relatively short half-life of ^68^Ga presents advantage in cases when repetitive examinations on the same day are of interest [85]. The high fraction of positron emission is another advantage of ^68^Ga (89%) as compared to ^64^Cu (19%) and ^124^I (23%). Comparison of some clinically used imaging agents demonstrates the lower effective dose that patient is exposed to when using ^68^Ga-based agent as compared to the agents comprising ^18^F, ^99m^Tc, and ^111^In (Table 2) [6, 86, 87]. Moreover, the duration of patient examinations is shorter for ^68^Ga-agents than that for SPECT agents, and to some extend for [^18^F]FDG. In summary, the use of ^68^Ga would be beneficial in terms of accessibility, high sensitivity and resolution, quantification, dynamic scanning, fast scanning protocol, repetitive examinations, and low radiation burden.

The chemical form in aqueous solution is Ga(III) cation which provides robust coordination chemistry. ^68^Ga-labelling can be direct or chelator mediated. The direct labelling utilizes the chelating ability of macromolecules, for example, lactoferrin and transferrin comprising Tyr, His, and Asp AA residues that can chelate Ga(III) in the presence of synergetic bicarbonate ion. Low molecular weight ligands can form stable complexes of variable lipophilicity and charge for nontargeting imaging. The chelator mediated ^68^Ga-labelling requires presence of a bifunctional chelator (BFC) for the subsequent, straightforward, and side specific coordination with Ga(III). Considerable number of chelators was successfully developed [4, 6, 88–95]. The most commonly used are DOTA and NOTA based chelators. The former requires heating under over 60°C for the complexation with ^68^Ga, while the latter can chelate ^68^Ga at ambient temperature which might be crucial in case of temperature sensitive ligands, and it also allows for cold kit type radiopharmaceutical preparation under radiopharmacy practice [96]. DOTA presents an advantage in the context of radiotheranostics since it can form stable complexes with ^68^Ga for PET diagnostics and ^177^Lu for radiotherapy.

The chelator or prosthetic group mediated labelling most commonly results in agents comprising biologically active vector molecule, chelator/prosthetic group moiety, and radionuclide. Very often pharmacokinetic modifiers (PKM) are incorporated in order to modulate pharmacokinetics and agent organ distribution and improve in vivo stability as well as separate the binding site from the bulky chelator/prosthetic group moiety which may deteriorate the biological activity of the vector molecule. Considerable number of publications reveal strong influence of even slight modifications in any of the agent structural components, and the accurate prediction of pharmacokinetics and pharmacodynamics of a new agent is not straightforward [97]. Nevertheless, vast experience and knowledge have been intensively gathered during last two decades providing possibility for more efficient and effective development. The labelling chemistry of ^68^Ga is well characterized and is relevant to small molecules, macromolecules, and particles.

Ga(III) as a chemical element presents a unique advantage over other radionuclides as it has properties closely resembling those of Fe(III) which is involved in many biochemical processes including inflammation. Moreover, Fe(III) is an essential nutrient and limiting factor of microbial life [98]. Stable Ga(III) has been used in treatment of various diseases including cancer, infection, and inflammation [99–101]. The ability of Ga(III) to bind iron proteins, for example, lactoferrin and transferrin as well as siderophores, and enzymes can be utilized in the imaging agent development.

## 4. Biomarkers and Radiopharmaceutical Development

The development of imaging agents relies strongly on the advances, experience, and knowledge of the research of biomarkers, for example, receptors and antigens; transport systems; substances involved in angiogenesis, glycolysis, hypoxia, proliferation, and apoptosis; and enzyme activity. Targeting biomarkers that are specific for a given disease is one the major aims of an agent development for both diagnostic imaging and therapy. The knowledge and access to respective vector molecules have considerably expanded due to the achievements in proteomics and genomics. Infection, inflammation, and fibrosis are closely interrelated processes and corresponding biomarkers might present practical interest in developing respective imaging agents. Favorable characteristics of a target in general include expression upregulation, absence of expression in normal tissue, and internalization or stable binding of the respective ligand for the longitudinal accumulation of the latter [102].

## 5. Imaging Inflammation

Inflammatory response is a complex process involving immune system cells (T- and B-lymphocytes, NK cells, macrophages, monocytes, neutrophils, eosinophils, and mast cells) and products of their (patho)physiological activity, for example, cytokines involved in the cell signaling. Various functions of the cells and their products as well as their receptors provide a broad range of potential imaging targets [103–107]. Targeting the white blood cells of the immune system such as macrophages, monocytes, lymphocytes, and neutrophils for the detection of their upregulation and trafficking, secretion of cytokines and chemokines, and phagocytosis has been investigated both clinically and preclinically. Receptors such as SSTR, NCA-90, integrins, folate, bombesin, vascular cell adhesion protein-1, and interleukins expressed by activated T-cells, CXCR2 expressed on neutrophils, and CXCR4 overexpressed by leukocytes have demonstrated potential for in vivo targeted imaging [108]. Respective ligands and substrates can be considered for radiolabelling. Cytokines including interferons, lymphokines, interleukins, and chemokines bind to various receptors, for example, IL1 and IL2 receptor types, IFN, CD40, CD37, CD30, CD4, CCR5, and IL1-17R receptor family. Folate, CD64, NCA90, and CD15 receptors expressed on macrophages, leukocytes, and granulocytes can serve as targets. Not only do molecules of such super families as chemokine, integrin, selectin, and immunoglobulin participate in the cell emigration cascade, but also enzymes on the surface of endothelia cells and leukocytes contribute to the leukocyte extravasation [109]. Receptors on the endothelial wall, for example, for binding of IL1 and TNF*α*, are another category of the targets. These are only few examples of targets for potential imaging agent development (Table 3). Many targets were utilized in oncology [28] and their translation to inflammation is feasible.

### 5.1. Targeting Cell Receptors with Antibodies

Radiolabelled (^99m^Tc, ^111^In, and ^123^I) anti-CD2, anti-CD5, anti-CD25, anti-CD45 antibodies and their fragments were used for the imaging of T-lymphocyte infiltration in various inflammatory diseases [110]. Typically for antibody slow pharmacokinetics, the time delay between the administration and examination stretches up to 24 hours. Interleukin-8 labelled with ^99m^Tc was studied in rabbits with induced acute pyogenic osteomyelitis [111] and induced acute colitis [112]. The agent was found suitable for the scintigraphic evaluation of the respective diseases. CD163 receptor expressed in monocytes and activated macrophages was targeted with an anti-CD163 antibody labelled with ^68^Ga in rats with acute collagen-induced arthritis [45]. The agent demonstrated specific binding and thus potential for studies of inflammatory diseases.

### 5.2. Targeting Angiogenesis

Angiogenesis plays an important role in wound healing, chronic inflammation, and tumour growth [113]. The family of vascular endothelial growth factors (VEGF) and integrins play crucial role in the angiogenesis cascade. Integrin receptors are overexpressed on the surface of vascular endothelial cells during angiogenesis in malignances, tissue healing, and inflammation. The largest group is radiolabelled peptide ligands comprising arginine-glycine-aspartic acid (RGD) sequence and peptidomimetics targeting *α*_v_*β*_3_ integrin receptors. Various analogues were developed introducing cyclization and multimerization; variety of chelate/coligand moieties; PKM such as carbohydrate and polyethylene glycol chains [114–121]. Various RGD analogues labelled with ^18^F, ^68^Ga, and ^99m^Tc were used in oncological clinical trials [122]. The majority of them comprised ^18^F; however, advantages of ^68^Ga such as accessibility of the radionuclide, more straightforward and efficient labelling chemistry, lower radiation dose, and better image contrast rendered more extensive development of ^68^Ga-based analogues [123–127].

The imaging agents tested in cancer systems can be relevant for the imaging of inflammation related diseases. The imaging and evaluation of synovial angiogenesis in patients with rheumatoid arthritis was accomplished using [^68^Ga]Ga-PRGD_2_ [46]. The elevated agent uptake was detected in the sites of active inflammation, rich neovasculature, and physiological integrin receptor expression, while no tracer accumulation was detected in axillary lymph nodes with reactive hyperplasia and strenuous skeletal muscles. [^68^Ga]Ga-PRGD_2_/PET-CT was found useful for the evaluation of synovial angiogenesis and follow-up of the treatment response.

[^68^Ga]Ga-NOTA-c(RGDyK) was developed for the imaging of myocardial infarction (MI) and follow-up of the response to the therapeutic intervention and demonstrated promising results preclinically [47]. The uptake in the MI lesions was enhanced and correlated with the vascular endothelial growth factor expression. Dynamic [^68^Ga]Ga-NOTA-c(RGDyK)/PET scanning with subsequent kinetic modeling studies in rats with forelimb ischemia showed higher uptake and distribution volume in the ischemic area as compared to that of sham operation and control regions [48]. Monitoring myocardial repair and angiogenesis after ischemic injury was found plausible using [^68^Ga]Ga-NODAGA-RGD and [^68^Ga]Ga-TRAP-(RGD)_3_ in rat model [49]. Elevated uptake of [^68^Ga]Ga-DOTA-E-[c(RGDfK)]_2_ was observed in the infarcted area while no accumulation was detected in the noninfarcted myocardium of the same rats [50]. The uptake of [^68^Ga]Ga-DOTA-RGD in atherosclerotic plaques was studied in vivo in atherosclerotic mice with promising results [52]. Elevated uptake of [^68^Ga]Ga-NODAGA-RGD in injured myocardium as compared to viable ischemic areas in pig model presumably indicated increased expression of *α*_V_*β*_3_ receptors associated with injury repair in the presence of coronary stenosis [51].

Although targeting VEGF receptors were studied in the context of cancerous diseases, chronic inflammation can also be considered. A ligand consisting of a single chain (scVEGF, 3–112 amino acids of human VEGF_121_) [128, 129] was labelled with ^68^Ga and the resulting agent showed distinct uptake in the tumour xenografts in mice; however high kidney uptake needed to be addressed [130, 131].

### 5.3. Targeting Selectins

P-selectin is expressed on the active endothelium surface and platelets and operates the migration of leukocytes in response to inflammatory cytokines. E-selectin binding peptide labelled with ^99m^Tc accumulated in acute osteomyelitic lesions in rats presumably by interaction with activated vascular endothelium [132]. An analogue of P-selectin natural ligand, fucoidan, labelled with ^68^Ga could discriminate active and inactive atheroschlerotic plaques in mice [44].

### 5.4. Targeting Vascular Adhesion Protein-1

**Figure 1:**
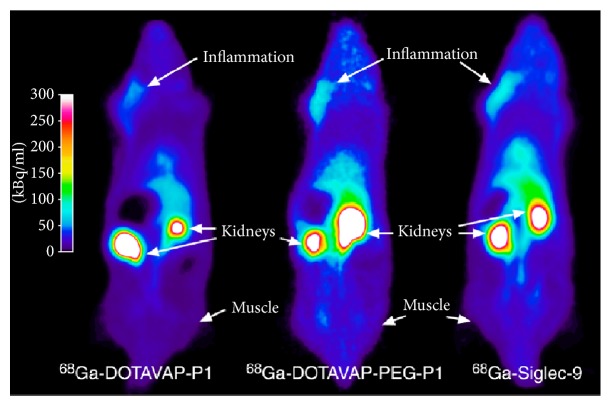
PET images of the distribution of [^68^Ga]Ga-DOTAVAP-P1, [^68^Ga]Ga-DOTAVAP-PEG-P1, and [^68^Ga]Ga-DOTA-Siglec-9 in turpentine-induced rat model of sterile inflammation. All three peptide analogues showed target-to-nontarget ratio above 6 with rapid accumulation in the inflammation site and renal clearance. Adapted from [57].

Vascular adhesion protein-1 (VAP-1) and CD73 are endothelial surface enzymes involved in the recruitment of leukocytes and their movement from the blood into the tissue [109]. Endothelial activation that takes place during inflammation can be utilized for specific targeting imaging. Several peptide analogues labelled with ^68^Ga were designed for the visualization of VAP-1 and showed promising results in animals with induced infection and sterile inflammation [7–13, 133]. The binding was proven specific and it was possible to differentiate inflammation from infection. [^68^Ga]Ga-Siglec targeting VAP-1 demonstrated preclinical potential for imaging of synovial inflammation in patients with rheumatic diseases [53]. The same agent was utilized for respiratory distress syndrome (ARDS, an inflammatory lung injury) imaging in a porcine model [54]. Imaging VAP-1 with [^68^Ga]Ga-Siglec was found promising also for the detection of inflamed atherosclerotic lesions [55] and inflammatory response induced by catheter implantation and staphylococcal infection [56]. ^68^Ga-Siglec and two more peptide analogues with affinity to VAP-1 ([^68^Ga]Ga-DOTAVAP-P1, [^68^Ga]Ga-DOTAVAP-PEG-P1, and [^68^Ga]Ga-DOTA-Siglec-9) were investigated in rat model of sterile skin/muscle inflammation (Figure 1) [57]. They showed distinct uptake in the affected sites.

### 5.5. Targeting Chemokines

Cytokines are produced by macrophages, B-lymphocytes, T-lymphocytes, and mast cells and act through receptors modulating, for example, immune response to infection and inflammation. Cytokines include chemokines, interleukins, interferons, and lymphokines that can be classified in broad families exhibiting diverse functions, for example, IL-1 and IL-6 superfamilies and TNF/TNF receptor superfamily. Therapeutics targeting cytokines are in clinical use, for example, inhibiting TNF or IL-6 in rheumatic diseases.

Chemokine receptors are physiologically expressed on B-lymphocytes, T-lymphocytes, macrophages, neutrophils, eosinophils, monocytes, and hematopoietic stem cells [134]. Imaging agents targeting CXCR4 are based on inhibitors (AMD3100) or small peptides (NFB, T140, pentixafor, and TN14003) and comprise ^18^F, ^67^Ga, ^68^Ga, or ^64^Cu [135–148]. They were developed and studied for the imaging of various cancerous diseases: lung, breast, prostate cancers, acute myeloid leukemia, and glioblastoma.

The application of CXCR4 targeting agents was extended beyond oncology. Clinical case/image reports [149, 150] were published on the utilization of [^68^Ga]Ga-pentixafor for detection and quantification of CXCR4 receptor density in ischemic heart diseases reflecting the role of the receptor in inflammatory and progenitor cell recruitment [58, 59]. The same agent was successfully used in the assessment of macrophage infiltration in atherosclerotic plaques in rabbit disease model [151].

### 5.6. Targeting Folate Receptors

**Figure 2:**
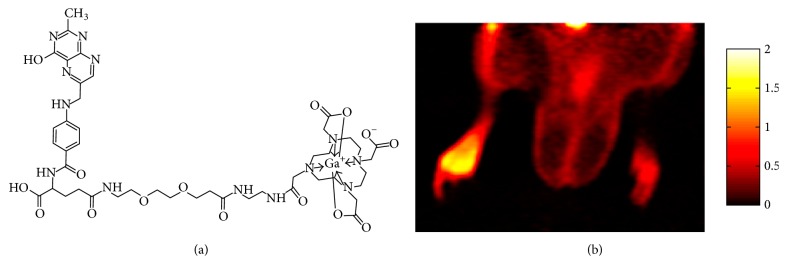
Accumulation of [^68^Ga]Ga-DOTA-folate (a) in the site of inflammation of rat inflammatory paw model induced by subcutaneously injected Complete Freund's Adjuvant (b). Adapted from [61].

Folate receptors (FRs) are overexpressed on a variety of cancer cells and activated macrophages, but not on normal cells [152, 153]. The enhanced expression of FR was found in lung macrophages during acute inflammation [154]. The majority of the nuclear imaging agents based on folic acid or pteroic acid [155] were developed for diagnosis of cancers overexpressing FR receptors such as breast, cervical, ovarian, colorectal, nasopharyngeal, renal, and endometrial cancers. Various ^68^Ga-labelled agents demonstrated accumulation in cell cultures and mice bearing folate-receptor positive human nasopharyngeal carcinoma cell line (KB) xenografts [6, 156–162]. [^68^Ga]Ga-DOTA-PEG-FA comprising folic acid was investigated for the detection and quantification of inflammatory response to medical implants using mice with subcutaneously implanted polylactic acid and poly(N-isopropylacrylamide) particles as a model [60]. The agent was accumulated in the area of the implant most probably reflecting interaction of [^68^Ga]Ga-DOTA-PEG-FA with folate receptor expressed on activated macrophages. Another folic acid based agent, [^68^Ga]Ga-DOTA-folate, was successfully tested in an inflammatory paw rat model (Figure 2) [61]. Distinct accumulation in inflamed hand and foot joints of rheumatoid arthritis of a ^99m^Tc-labelled folate analogue was observed in a patient, while no uptake was detected in a nonarthritis patient's hands and feet [163].

### 5.7. Targeting Somatostatin Receptors

Somatostatin receptor (SSTR) ligand analogues have found an extensive application in diagnosis and radiotherapy of neuroendocrine tumours. The elevated expression of SSTRs is known also in small cell lung cancer, breast cancer, renal cell carcinoma, prostate cancer, and malignant lymphoma. A number of somatostatin ligand analogues labelled with gamma- and positron-emitting radionuclides were used clinically for oncological cases [85, 164–174]. ^68^Ga-labelled somatostatin analogues demonstrated superior performance in terms of higher specificity and sensitivity, detection rate, shorter examination time, and quantification possibility and have become a golden standard for the detection of neuroendocrine tumours (NETs) taking over that title from [^111^In]-pentetreotide (OctreoScan®) and demonstrating specificity and sensitivity of over 90% [27, 175–180]. ^68^Ga-labelled agents for the imaging of NETs demonstrated advantages also over other radionuclides and tracers such as [^18^F]FDG [174], ^123^I-metaiodobenzylguanidine ([^123^I]MIBG) [181, 182], [^18^F]DOPA [183], [^99m^Tc]-dicarboxy propane diphosphonate [184], and [^18^F]NaF.

**Figure 3:**
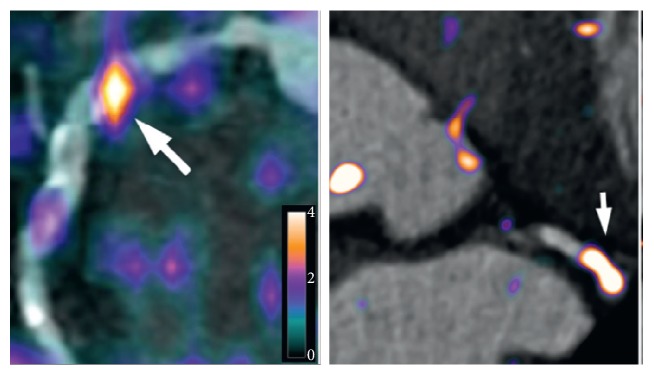
Intense atherosclerotic inflammation (white arrows) was detected by [^68^Ga]Ga-DOTA-TATE in a patient with acute coronary syndrome. Adapted from [65].

SSTR are also overexpressed on activated macrophages and T-lymphocytes. ^68^Ga-labelled analogues were used in inflammation related diseases such as idiopathic pulmonary fibrosis [62], Graves' and Hashimoto's diseases [63], coronary artery plaque imaging and characterization [64], and atherosclerotic inflammation with excellent macrophage specificity (Figure 3) [65]. Promising diagnostic potential of a ^99m^Tc-labelled analogue was demonstrated in patients with rheumatoid arthritis and secondary Sjogren's syndrome, and the method was suggested for the assistance in anti-TNF alpha antibody treatment planning [185]. [^68^Ga]Ga-DOTA-TOC/PET-CT was found superior to ^67^Ga-Citrate/SPECT in detection of sarcoidosis lesions [186]. A clinical study demonstrated correlation between uptake of [^68^Ga]Ga-DOTA-TOC and SST_2_ mRNA expression and recorded the information in a database [187] providing tools for accurate quantification and evaluation of disease progression and treatment response in cancerous and inflammatory diseases involving SSTRs. Preclinical study using atherosclerotic mice demonstrated superior targeting properties of [^68^Ga]Ga-DOTA-NOC as compared to [^18^F]FDR-NOC [188], overall confirming the potential of SSTR targeting for atherosclerotic plaque imaging.

### 5.8. Imaging Neuroinflammation

Despite difficulty of designing ^68^Ga-labelled molecules capable of blood-brain barrier penetration, several agents were suggested for the imaging of neuroinflammation, in particular A*β* plaques deposited on blood vessels [67–69]. Bivalent styrylpyridines labelled with ^68^Ga demonstrated high specificity and affinity for A*β* plaques using postmortem Alzheimer's disease (AD) brain sections [67]. Benzofuran derivative comprising ^68^Ga showed promising results in terms of binding specificity and affinity investigated in vitro in sections of Tg2576 mice [68]. Although the synthesis of a ^68^Ga-labelled Pittsburgh compound analogue was successful, the in vitro binding to amyloid deposits was limited [69]. The common disadvantage of these agents is poor blood-brain barrier penetration; nevertheless the exploration of more successful analogues continues. Curcumin functions as an antioxidant, antimicrobial, anti-inflammatory, and anticancer agent. Diacetyl-curcumin and bis(dehydroxy)curcumin labelled with ^68^Ga demonstrated in vitro binding to *β*-amyloid fibrils and lung cancer cells [189]. Potential application of the agents could include diagnostic imaging of Alzheimer's disease and various cancers.

## 6. Imaging Infection

Infection imaging can be indirect utilizing targets involved in the immune response, namely, inflammation, as presented in the inflammation targets section above or direct utilizing pathogen related targets. The direct imaging is especially crucial in cases where inflammatory response is absent. The difference in biochemistry and structure between bacterial and human cells might exclude physiological uptake by human tissue making it easier to meet the favorable characteristics of an imaging agent. However, discrimination between the various infectious microorganisms, pathogenic bacteria, and microbiota, targeting specific bacteria type as well as difficulty of accessing bacteria aggregated in a biofilm makes the task very challenging [190, 191]. The specific targeting of infection would require accumulation of the radioactive signal in the pathogen. The radiolabelled targeting agents for infection can be roughly divided into several groups: antibiotics based; antimicrobial protein and peptide based; siderophore and other metabolisable compound based; and antigen-specific antibodies and antibody fragments (Table 3).

### 6.1. Radiolabelled Antibiotics

Antimicrobials act on the processes that are specific to microbes, for example, bacteria and fungi, and thus corresponding imaging agents might distinguish infection from inflammation [191]. They might require internalization or may bind to the cell surface dependent on their biological action mechanism [191–193]. The possibility of antibiotic resistance development exists also in the case of imaging agents even though the amount of such agents would be subnanomolar [194, 195]. Another complication is possible nonspecific uptake of antibiotics based agents by leucocytes [196]. Considerable number of various antibiotic analogues have been labelled with ^99m^Tc, ^111^In, ^131^I, ^11^C, and ^18^F [102] and evaluated preclinically and clinically with ^99m^Tc-ciprofloxacin becoming a commercial product (Infecton) [21, 197, 198]. However, the further improvement of specificity is desirable [191]. Antibiotics are accessible and cheap, and they demonstrate high sensitivity [102, 191] making the development of ^68^Ga-labelled analogues very attractive given the earlier mentioned advantages that ^68^Ga as a radionuclide in combination with PET provides. Two ^68^Ga-labelled analogues based on ciprofloxacin demonstrated potential for discrimination between bacterial infection and inflammation in rats infected with* Staphylococcus aureus* [70].

### 6.2. Radiolabelled Antimicrobial Proteins and Peptides

Antimicrobial proteins and peptides, for example, serprocidins, cathelicidins, and defensins produced by the cells of immune system, target microbial membrane lipids and impose microbicidal effect [35, 43]. They present a large group of potential candidates for microbial imaging including bacteria, fungi, parasites, and viruses. Antimicrobial peptides have demonstrated higher specificity for infection than antibiotic analogues. They accumulate at infection but not sterile inflammation sites. The most thoroughly studied antimicrobial peptide, ubiquicidin UBI [29–41] labelled with ^99m^Tc [199], demonstrated promising results in human clinical trials [200, 201]. It has the potential for quantification of viable infecting microorganisms and consequently for monitoring the efficacy of antimicrobial therapy in patients.

**Figure 4:**
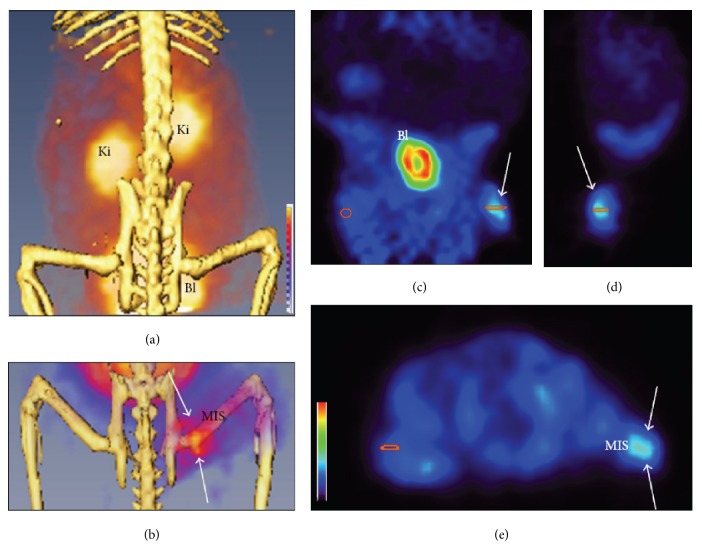
Left panel presents maximum intensity projection images of [^68^Ga]Ga-DOTA-TBIA101 distribution in a healthy mouse (a) and a mouse with muscular infection site (MIS) in the right hind muscle tissue (white arrows). Right panel presents coronal (c), sagittal (d), and axial (e) images with uptake in the MIS (white arrow) and absence of the uptake in the contralateral muscle tissue. Ki and Bl stand, respectively, for kidney and bladder. Reproduced from [73].

Fragments of an antimicrobial peptide ubiquicidin conjugated to NOTA and labelled with ^68^Ga, [^68^Ga]Ga-NOTA-UBI29-41, and [^68^Ga]Ga-NOTA-UBI30-41 demonstrated possibility for the distinction between infection and inflammation in a rabbit model [71, 72]. Antimicrobial peptide fragments GF-17 and RAWVAWR-NH2 of, respectively, human cathelicidin LL-37 and human lysozyme active against* E. coli* and* S. aureus* were labelled with ^68^Ga and their biodistribution in normal rats demonstrated fast clearance from liver [75]. Antimicrobial depsipeptide based agent, [^68^Ga]Ga-DOTA-TBIA101, targeting bacterial lipopolysaccharides detected muscular* E. coli*-infection in mice (Figure 4) [73]. The agent was also studied in healthy rabbits and various disease model rabbits such as sterile inflammation,* Staphylococcus aureus* infection, and* Mycobacterium tuberculosis* [74]. The clearance of [^68^Ga]Ga-DOTA-TBIA101 from blood and normal tissue was fast, and enhanced uptake in sterile inflammation and* Mycobacterium tuberculosis* sites was observed. The improvement of the bacterial selectivity will require modification of the agent structure.

### 6.3. Radiolabelled Siderophores

Bacteria and fungi produce various siderophores for harvesting iron which is essential for their survival and growth [34, 98, 191]. Siderophores can also play a critical role in the development of biofilms by microbes. They are low molecular weight compounds specifically chelating Fe(III), and Ga(III) can form stable complexes with them mimicking Fe(III) [202, 203].

**Figure 5:**
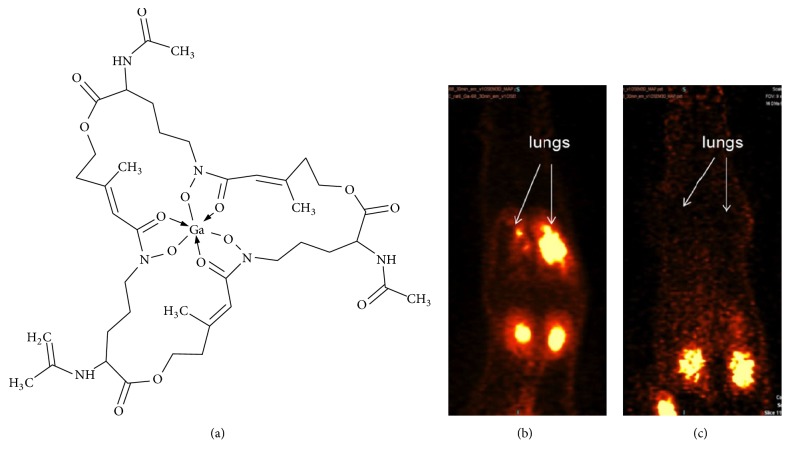
Molecular structure of [^68^Ga]Ga-triacetylfusarinine C (a) used for the in vivo imaging of a rat with Aspergillus* fumigatus *infection (b) and negative control of noninfected rat (c). White arrows point at the infected (b) and normal (c) lungs. Adapted from [76].

Desferri-triacetylfusarinine C (TAFC) and desferri-ferricrocin (FC) labelled with ^68^Ga were used for the imaging of invasive pulmonary aspergillosis (IPA) caused by Aspergillus* fumigatus *[15]. [^68^Ga]Ga-TAFC demonstrated superior characteristics in terms of specific target binding, metabolic stability, and fast blood clearance in a rat model of* A. fumigatus *infection. Seven analogues were developed in another study with TAFC and ferrioxamine E (FOXE) showing favorable binding, clearance, elimination, and stability characteristics [16] as well as lung uptake in rat of invasive aspergillosis model wherein the uptake extent was correlated with disease severity [17]. [^68^Ga]Ga-triacetylfusarinine C and [^68^Ga]Ga-ferrioxamine E were investigated in rat model of* A. fumigatus* and demonstrated rapid uptake in the lungs (Figure 5) [76].

### 6.4. Radiolabelled Metabolisable Agents

Mammalian microbiota consumes (poly)saccharides, in particular maltose and maltodextrins [204]. The transport mechanism is specific to bacteria and is absent in mammalian cells making it possible to utilize these (poly)saccharides for imaging agent development. Maltodextrin functionalized with a fluorescent dye was internalized through the bacteria-specific maltodextrin transport pathway and discriminated between active bacteria and inflammation in vivo [192]. Maltose labelled with ^18^F localized specifically bacterial infection in mice [205]. Potential to label polysaccharides directly with ^68^Ga might be utilized extensively.

As mentioned above, the chemical properties of Ga(III) provide the potential for direct labelling of polysaccharides. Dextran was labelled directly and resulting complex demonstrated sufficient stability in human serum; however the feasibility of the bacterial imaging was not demonstrated [206].

Trapping of nucleosides that are substrates of thymidine kinase occurring within bacteria was explored using ^18^F and ^125^I labelled analogues of uracil [207]. Promising results were obtained in seven bacterial species in mice. Another study, in the context of therapeutic bacteria development, demonstrated possibility of detecting* Salmonella* vectors within tumours using ^18^F-labelled uracil [208]. However, the development of ^68^Ga-labelled nucleosides that would maintain their biological activity is challenging and few examples known from the literature confirm that [4, 6].

## 7. ^68^Ga-Citrate

**Figure 6:**
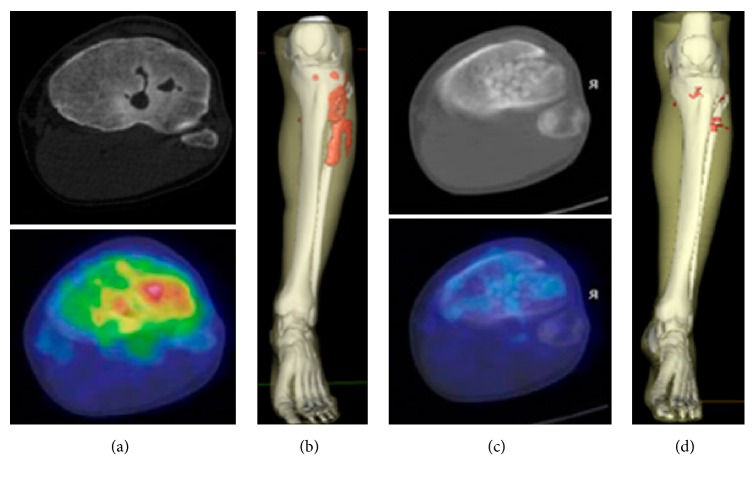
[^68^Ga]Ga-citrate PET/CT examination of a patient affected by acute osteomyelitis before (left panel) and after (right panel) surgical curettage showing uptake in the transaxial (a, c) and 3D reconstruction images (b, d; red area). Absence of the uptake after the therapy confirms complete response to the treatment. Adapted from [19].

**Figure 7:**
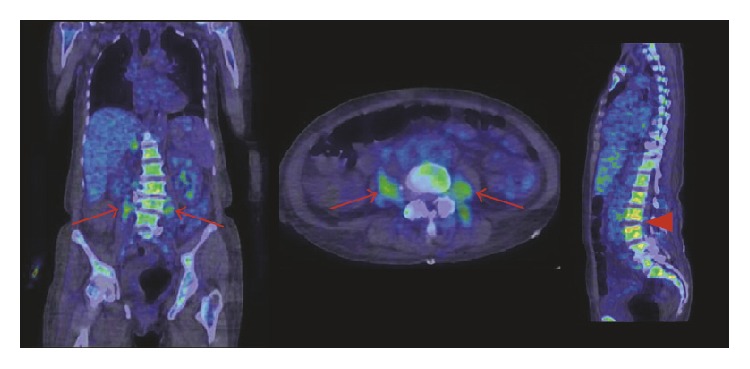
Vertebral osteomyelitis (spondylodiscitis; red arrowheads) and abscesses in the iliopsoas and paravertebral area (red arrows) were detected by [^68^Ga]Ga-citrate in a patient admitted to the hospital with back pain and general symptoms. The PET acquisition was performed 88 min after administration of 245 MBq of [^68^Ga]Ga-citrate. Adapted from [80].

As mentioned above ^68^Ga/PET provides a number of advantages over ^67^Ga/SPECT and following publications demonstrate it in clinical and preclinical studies. [^68^Ga]Ga-citrate demonstrated high diagnostic accuracy of 90% of osteomyelitis and diskitis in clinical studies (Figure 6) [18, 19]. This study demonstrates that [^68^Ga]Ga-citrate can be employed for monitoring the response to treatment. [^68^Ga]Ga-citrate was used clinically to follow-up surgical intervention in patients with acute osteomyelitis and intra-abdominal infection [77]. The agent was also used to successfully visualize lung malignancy and tuberculosis in patients; however in case of high prevalence of granulomatous diseases the distinction between malignant and benign lung lesions was unclear [78, 79]. Another clinical study conducted head-to-head comparison of [^68^Ga]Ga-citrate (Figure 7) and [^18^F]FDG in patients with* Staphylococcus aureus* bacteremia [80]. The detection rate of osteomyelitis was similar, and further investigation of [^68^Ga]Ga-citrate applicability in cases of osteomyelitis induced by other pathogens as well as for monitoring healing process is warranted.

Comparative study of [^68^Ga]Ga-citrate and [^67^Ga]Ga-citrate was performed in healthy and infection model rats [77]. The performance of [^68^Ga]Ga-citrate was found superior in terms of image contrast in the lower abdomen and extremities. Potential of [^68^Ga]Ga-citrate for the differentiation of acute interstitial nephritis from acute tubular necrosis was studied in rat model of the disease and it was demonstrated that the kidney uptake correlated with the extent of mononuclear cell infiltration accompanying inflammation [209]. ^68^Ga*-*labelled* Apo*-transferrin demonstrated bacterial infection detection capacity in rat model with* Staphylococcus aureus* wherein the infection site was visualized 1 h after administration of the agent [14].

### 7.1. Radiolabelled Antibodies and Antibody Fragments

Human immunoglobulin (HIG) binds to bacteria but also accumulates at the sites of fungal and viral infection as well as sterile inflammation due to binding to leukocytes. The improved specificity for bacteria was achieved for the fragments of HIG. It is feasible to develop specific antibodies to various antigens present on the bacterial cell surface [102]. Monoclonal antibodies labelled with ^99m^Tc were used for infection imaging via granulocytes targeting NCA-95 [210]. Various cytokines of interleukin family (IL-1, IL-8) labelled with ^123^I or ^99m^Tc demonstrated accumulation in the sites of infection in various animal models [111, 112, 211–214]. Registered antigranulocyte radiopharmaceuticals such as LeuTech®, Scintimun®, and Leukoscan® are based on ^99m^Tc-labelled antibodies. This experience can be translated to ^68^Ga; however either the size of the antibodies must be reduced or pretargeting techniques must be applied in order to overcome the discrepancy between the short physical half-life of ^68^Ga and slow pharmacokinetics of antibodies.

### 7.2. Radiolabelled Biotin

Biotin is a growth factor utilized in many bacteria. An ^111^In-labelled analogue of biotin was successfully utilized for diagnosis of vertebral infections in a clinical study [215]. It would be rational to explore the relevance of ^68^Ga-labelled analogues given the advantages of ^68^Ga over ^111^In and promising [^68^Ga]Ga-DOTA-Biotin analogues [216, 217] developed for monitoring survival of transplanted avidin-coated islets.

## 8. Miscellaneous

Stable Ga(III) complex with thiosemicarbazones demonstrated antimicrobial effect against* P. aeruginosa* and* C. albicans *due to most probably both displacement of essential Fe(III) with Ga(III) and thiosemicarbazones [101]. Substitution of the stable Ga(III) by radioactive ^68^Ga might result in a specific infection imaging agent.

Selective imaging of Enterobacteriaceae using 2-[^18^F]-fluorodeoxysorbitol (^18^F-FDS) was demonstrated in a murine myositis model [218]. The uptake of ^18^F-FDS was correlated with bacterial burden; moreover the agent differentiated infection from sterile inflammation. Given the potential of ^68^Ga for the labelling of small biologically active molecules [4] it might be plausible to develop a respective analogue with added value of the advantages that ^68^Ga offers including simpler production chemistry, lowered radiation dose, repetitive examination, and accessibility at clinical centers without cyclotrons and remote from [^18^F]-FDG distribution sites. As mentioned above, the poor access to bacteria aggregated in a biofilm might make the imaging task challenging. Several peptide candidates with affinity for S. aureus biofilm were designed and labelled with ^68^Ga [219]. The resulting agents demonstrated binding in vitro; however it was not possible to block the binding with excess of the cold peptide.

Ionic ^68^Ga was found superior to [^18^F]-FDG in infection detection in the rat model with diffuse osteomyelitis [220]. In another study, the uptake of ionic ^68^Ga was observed in the aortic plaques of atherosclerotic mice, specifically at the sites rich in macrophages [221]. However, the slow blood clearance of ionic ^68^Ga presents a limitation.

Chronic inflammation is the major reason of fibrosis [222]. ^68^Ga-labelled SST analogue ([^68^Ga]Ga-DOTA-NOC) demonstrated uptake in pathogenic areas in patients affected by idiopathic pulmonary fibrosis with potential for monitoring response to treatment and drug development [62]. Another clinical study using [^68^Ga]Ga-pentixafor also showed potential of the agent for monitoring disease activity and response to treatment in idiopathic pulmonary fibrosis [223]. Peptide based agents, CNO2A-PEG_2_-c[CPGRVMHGLHLGDDEGPC] and [^68^Ga]Ga-NODAGA-PEG_2_-c[CPGRVMHGLHLGDDEGPC] for the imaging and quantification of fibrosis by PET were developed and characterized preclinically showing fast clearance from normal tissue and blood and binding specificity [89]. Dosimetry calculations demonstrated possibility of six examinations per year in humans assuring disease monitoring in longitudinal studies and routine clinical setup [224].

Several hyaluronan conjugates of oligonucleotides targeting CD44 positive cells were developed and tested in healthy rats, sham-operated rats, and rats with myocardial infarction [225]. The uptake of the agents was higher for the latter group and varied dependent on the difference in the oligonucleotide structure.

TLR2 and TLR4 expression levels in neutrophils were found higher in individuals with bacterial and viral infections than those in control samples. There is a possibility that IL-4, IL-8, IL-10, IL-12, and TNF-a might serve as biomarkers for infections and that IL-2, IL-8, or IL-10 is potentially able to distinguish between bacterial and viral infections [22].

Mannosylated human serum albumin labelled with ^68^Ga via NOTA chelator moiety ([^68^Ga]Ga-NOTA-MSA) was tested in a rat model of myocarditis targeting mannose receptors expressed on macrophages infiltrating myocardium [66]. The uptake in the diseased myocardium was considerably higher than that of the normal one and it was precluded by administration of excess of nonlabelled MSA indicating binding specificity. The tracer build-up was also observed in the organs of macrophage accumulation.

[^68^Ga]Ga-DOTA was investigated for the quantification of increased blood flow which is one of the key events in inflammation [226]. The uptake kinetics of [^68^Ga]Ga-DOTA in the site of inflammation in rats with induced inflammation correlated well with that of ^15^O-water, suggesting high relevance [^68^Ga]Ga-DOTA.

## 9. Pretargeted Imaging

The half-life of ^68^Ga is shorter than that of ^64^Cu, ^67^Ga, ^99m^Tc, ^89^Zr, ^111^In, and ^123,124,125^I and thus in contrast to the latter it is not compatible with slow pharmacokinetics of large molecules such as antibodies and glycoproteins. The range of antigen-specific antibodies relevant to inflammation and infection is broad and a number of ^99m^Tc-labelled antibodies were used clinically [20, 21, 227]. The respective range of ^68^Ga-based agents could be similar. The solution to overcome the incompatibility of half-life time frames could be either the reduction of the antibody size or the application of the pretargeting concept.

**Figure 8:**
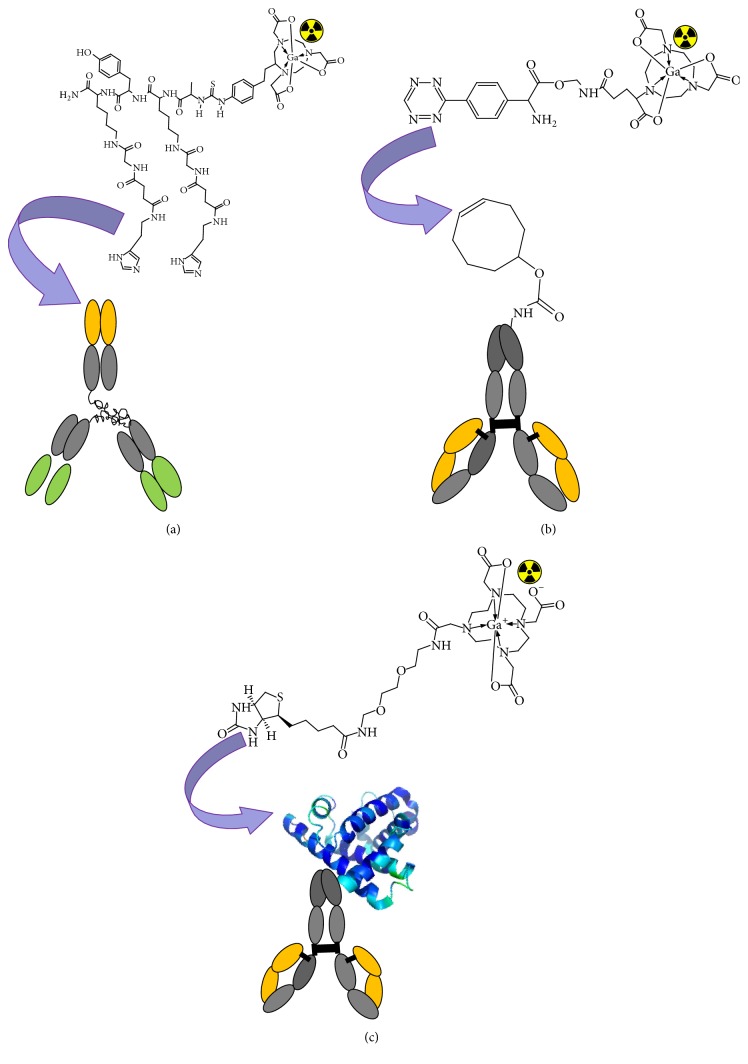
Schematic presentation of pretargeting techniques: (a) bispecific antibodies engineered to specifically bind with radiolabelled hapten molecules; (b) bioorthogonal click chemistry for fast and specific covalent binding between, for example, a* trans*-cyclooctene functionalized antibody and a radiolabelled tetrazine; (c) interaction between antibody-(strept)avidin conjugate and radiolabelled biotin utilizing extremely high affinity of (strept)avidin and biotin.

The history of the pretargeting concept spans three decades, predominantly in the field of oncology [228–230]. It was developed to improve image contrast and dosimetry in immunoimaging and radioimmunotherapy when using radiolabelled antibody ligands with slow pharmacokinetics [231]. The arsenal of antibodies is vast and diverse encouraging extensive investment into development of techniques that would allow their exploration to the fullest. Pretargeting considers at least two major steps wherein a functionalized antibody is first administered for target localization and clearance from blood and normal tissue and thereafter a radiolabelled small molecule capable of binding to the functionalized antibody due to high affinity or covalent interaction is administered. The key properties of the radiolabelled molecules are fast pharmacokinetic and clearance. Several techniques have been developed for the realization of pretargeting concept including avidin/streptavidin-biotin systems [216, 217, 232, 233]; bispecific antibodies (bsmAb) with haptens [232, 234–254]; antibody-oligonucleotide conjugates with complementary oligonucleotides [255]; biorthogonal systems allowing covalent chemical reactions in vivo (Figure 8).

The high affinity of biotin to avidin and streptavidin proteins was utilized clinically and preclinically in pretargeting approach for the imaging and therapy of pancreatic adenocarcinoma [232], glioblastoma [256], and lymphoma [257]. However, this pretargeting technique may require three steps in order to eliminate the excess of antibody-(strept)avidin conjugate, circulating in the blood and not bound to the target, by adding clearing agent. Another application of the technique was monitoring transplantation of islets of Langerhans in the treatment for type 1 diabetes mellitus, wherein the cells or cell mimetics were conjugated to (strept)avidin prior to the transplantation [216, 217]. Several analogues of biotin comprising DOTA chelate moiety for labelling with ^68^Ga and ethylene glycol linker of various length demonstrated the influence of the latter on the affinity towards avidin.

Particular example of hapten molecules is the ones comprising histamine-succinyl-glycine (HSG) motif and chelate moiety [251–253, 258] for the complexation with ^68^Ga. Several analogues were developed for the imaging of carcinoembryonic antigen (CEA) pretargeted with anti-CEA bsmAb [254, 259, 260], and two clinical studies of medullary thyroid carcinoma and breast carcinoma positive for CEA using ^68^Ga-labelled hapten molecules and bsmAb were initiated [261].

Bioorthogonal reactions are fast, regioselective, requiring small reagent concentration, and occurring under mild conditions often in aqueous solution and temperature below 37°C [262, 263]. Amongst various biorthogonal reaction types, the cycloaddition of tetrazines and various dienophiles referred to as inverse-electron-demand Diels-Alder (IEDDA) reaction is the most successful in the context of pretargeting. Antibodies functionalized with* trans*-cyclooctene (TCO) and a radiolabelled tetrazine that can interact in vivo based on IEDDA reaction were studied [264–267]. In particular, ^68^Ga-labelled tetrazine dextran demonstrated favorable pharmacokinetics in a healthy mouse [264]. However, the proof of concept is to be performed in a xenografted animal. Accumulation of anti-TAG72 [265] and anti-A33 [266] antibodies functionalized with TCO in mouse xenografts was visualized, respectively, by an ^111^In and ^64^Cu-labelled tetrazine analogues. Anti-CA19.9 antibody-TCO in combination with ^177^Lu-labelled tetrazine demonstrated radiotherapeutic effect in pancreatic cancer murine model [267].

The pretargeted imaging techniques may contribute to the expansion of immuno-PET with ^68^Ga providing the intrinsic advantages of ^68^Ga and PET. As mentioned above, most of the developed radiolabelled counterparts of pretargeting techniques have demonstrated promising results. There are a considerable number of potential antibody biomarkers that could be considered for the imaging of infection and inflammation.

## 10. Theranostics Potential

Theranostics [268] embraces realization of personalized medicine by conducting diagnosis on individual basis and providing possibility of predicting the efficacy of a specific treatment and following up the response to the treatment enabling adjustment of the latter very early in the process. In the context of nuclear medicine wherein the radiopharmaceuticals targeted at biomarkers specific to a disease can carry either diagnostic radionuclides or therapeutic ones, the concept can be denoted as radiotheranostics [28]. The targeted molecular imaging such as PET can offer noninvasive diagnosis specific to the disease, for example, tumour-type specific, and provide accurate localization of the lesions. The strongest advantage of PET is the potential for quantification of the target, for example, receptor expression, investigation of the uptake kinetics, and estimation of the dosimetry. These characteristics of PET allow for individualized treatment selection and planning, monitoring of treatment response, and detection of recurrent disease. The individualized patient management provides such advantages as optimization of the treatment regimen for the improved response and exclusion of futile treatments, minimization of risks and toxicity with overall outcome of reduced cost and patient distress. The importance of individualized patient management was demonstrated by clinical studies wherein the influence of dose of the administered radiopharmaceutical, targeted at receptors overexpressed in cancer lesions, on the diagnostic outcome was investigated in the same patient [85, 269, 270]. ^68^Ga-labelled SST analogues [26–28, 271] and Affibody molecules [5, 272–274] used, respectively, in NENs and breast cancer patients are the most prominent examples of (radio)theranostics involving ^68^Ga/PET wherein ^68^Ga-labelled analogues were used not only for localization of the lesions, but also for staging, patient stratification, prognosis, therapy selection, and monitoring of the response to the treatment of NETs and other cancer types [2–4, 6, 85, 176, 275–277].

The methodology can be translated to inflammation and infection allowing for accurate and specific selection of treatment regimen and for follow-up and evaluation of the response to therapy, resulting in improved treatment efficacy and decreased cost and side effects. The accommodation of both imaging function and antibiotic function in the same molecule is a novel example of a theranostic agent [278]. A series of siderophores conjugated with DOTA moiety for the radiolabelling and with antibiotics for the treatment of bacterial infection were investigated preclinically. The accumulation of the intravenously administered ampicillin conjugate in the site of subcutaneously injected* P. aeruginosa* in mice was clearly and focally visualized within 0.6 h with retention for at least 24 h. These results obtained using analogues carrying dye for optical imaging can be translated to ^68^Ga-labelled counterparts for PET.

## 11. Conclusions

The medical need for specific agents for noninvasive, quantitative, and whole-body imaging of inflammation and infection has not been met yet despite decades of research. However, the prerequisites in terms of identification of potential targets, design and synthesis of the respective ligands, and imaging technologies are evolving very fast. The potential of accurate and quantitative lesion localization as well as monitoring of the treatment response promises personalized patient management.

The use of ^68^Ga in oncology is established proving the strong potential of ^68^Ga for the promotion of PET technology for effective and efficient diagnostics and personalized medicine. The experience of oncological ^68^Ga-based agents is getting translated to inflammation and infection. Pretargeted imaging technology opens wide possibilities based on antibody biomarkers.

## Figures and Tables

**Table 1:** Positron-emitting, gamma-emitting, and therapeutic radionuclides, their physical characteristics, and production mode. Adapted from [4].

Radionuclide	Half-life	*E*_max_ (keV)	Radiation	Production
*Positron emitters*				
^18^F	110 min	634	*β* ^+^ (97%)	Accelerator
^64^Cu	12.8 h	656	*β* ^+^ (19%)	Accelerator
^68^Ga	67.6 min	1899, 770	*β* ^+^ (89%)	Generator
^89^Zr	78.4 h	900	*β* ^+^ (23%)	Accelerator
^124^I	4.17 d	2100	*β* ^+^ (23%)	Accelerator

*Gamma emitters*				
^67^Ga	78.26 h	91, 93, 185, 296, 388	*γ*	Accelerator
^99m^Tc	6.0 h	141	*γ*	Generator
^111^In	67.9 h	245, 172 (0.5–25)	*γ*, Auger electrons	Accelerator
^123^I	13.3 h	159	*γ*	Accelerator

*Therapeutic radionuclides*				
^177^Lu	6.71 d	113, 208.4 (598)	*γ* (*β*^−^)	Reactor

**Table 2:** Effective doses for some PET and SPECT imaging agents. Reproduced from [6].

Agent	Examination time	Effective dose, [mSv]
[^111^In]In-DTPA-octreotide/SPECT	24–48 h	10.8
[^68^Ga]Ga-DOTA-TOC/PET	30–60 min	2.3
[^18^F]FDG/PET	60–120 min	5.6
[^99m^Tc]-BPAMD/SPECT	2–6 h	6
[^99m^Tc]-MDP/SPECT	2–6 h	3-4
[^68^Ga]Ga-BPAMD/PET	30–60 min	3-4

**Table 3:** ^68^Ga-based imaging agents for inflammation and infection investigated preclinically and clinically.

Target/mechanism	Imaging agent	Disease/microorganism (study type)
	*Inflammation*	

P-selectin	[^68^Ga]Ga-Fucoidan	Atherosclerotic plaques (preclinical [44])

Anti-CD163	[^68^Ga]Ga-anti-CD163-antibody	Acute collagen-induced arthritis (preclinical [45])

Integrins	[^68^Ga]Ga-PRGD_2_	Rheumatoid arthritis (clinical [46])

Integrins	[^68^Ga]Ga-NOTA-c(RGDyK) [^68^Ga]Ga-NODAGA-RGD [^68^Ga]Ga-TRAP-(RGD)_3_ [^68^Ga]-DOTA-E-[c (RGDfK)]_2_	Myocardial infarction (preclinical [47–51])

Integrins	[^68^Ga]Ga-NODAGA-RGD	Atherosclerotic plaques (preclinical [52])

VAP-1	[^68^Ga]Ga-Siglec	Synovial inflammation; inflammatory lung injury; atherosclerotic lesions; skin/muscle inflammation (preclinical [53–56])

VAP-1	[^68^Ga]Ga-DOTAVAP-P1, [^68^Ga]Ga-DOTAVAP-PEG-P1	Skin/muscle inflammation (preclinical [57])

CXCR4	[^68^Ga]Ga-pentixafor	Ischemic heart; atherosclerotic plaques (clinical [58, 59])

FR	[^68^Ga]Ga-DOTA-PEG-FA [^68^Ga]Ga-DOTA-folate	Inflammation/implant (preclinical [60, 61])

SSTR	[^68^Ga]Ga-DOTA-TOC	Sarcoidosis, idiopathic pulmonary fibrosis, Graves' disease, Hashimoto's disease, coronary artery plaque, atherosclerotic inflammation (clinical [62–65])

Mannose receptors	[^68^Ga]Ga-NOTA-MSA	Myocarditis (preclinical [66])

A*β* plaques	^68^Ga-labelled styrylpyridines, benzofuran, curcumin	Neuroinflammation, Alzheimer's disease (preclinical [67–69])

	*Infection*	

Antibiotics/inhibitor	[^68^Ga]Ga-ciprofloxacin	*Staphylococcus aureus* (preclinical [70])

Antimicrobial/membrane	[^68^Ga]Ga-NOTA-UBI29-41 [^68^Ga]Ga-NOTA-UBI30-41	*Staphylococcus aureus* (preclinical [71, 72])

Antimicrobial/membrane	[^68^Ga]Ga-DOTA-TBIA101	*E. coli* (preclinical [73, 74])

Antimicrobial/membrane	[^68^Ga]Ga-GF-17 and [^68^Ga]Ga-RAWVAWR-NH2	*E. coli* and *S. aureus* (preclinical [75])

Siderophores	[^68^Ga]Ga-TAFC, [^68^Ga]Ga-FC, [^68^Ga]Ga-FOXE	Invasive pulmonary aspergillosis (preclinical [15, 16, 76])

Leukocytes	[^68^Ga]Ga-citrate	Osteomyelitis, diskitis, intra-abdominal infection, tuberculosis, interstitial nephritis (clinical [18, 19, 77–80])

Leukocytes	[^68^Ga]Ga-Apo-transferrin	*Staphylococcus aureus* (preclinical [14])
